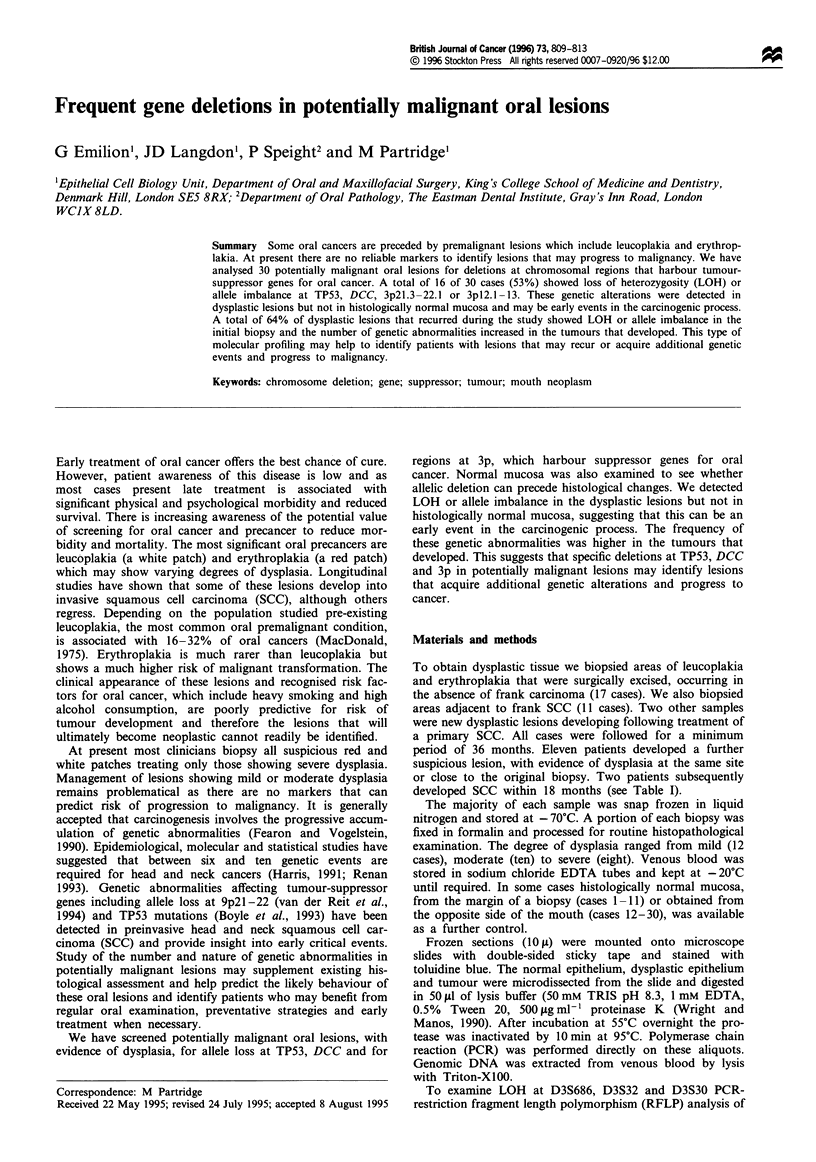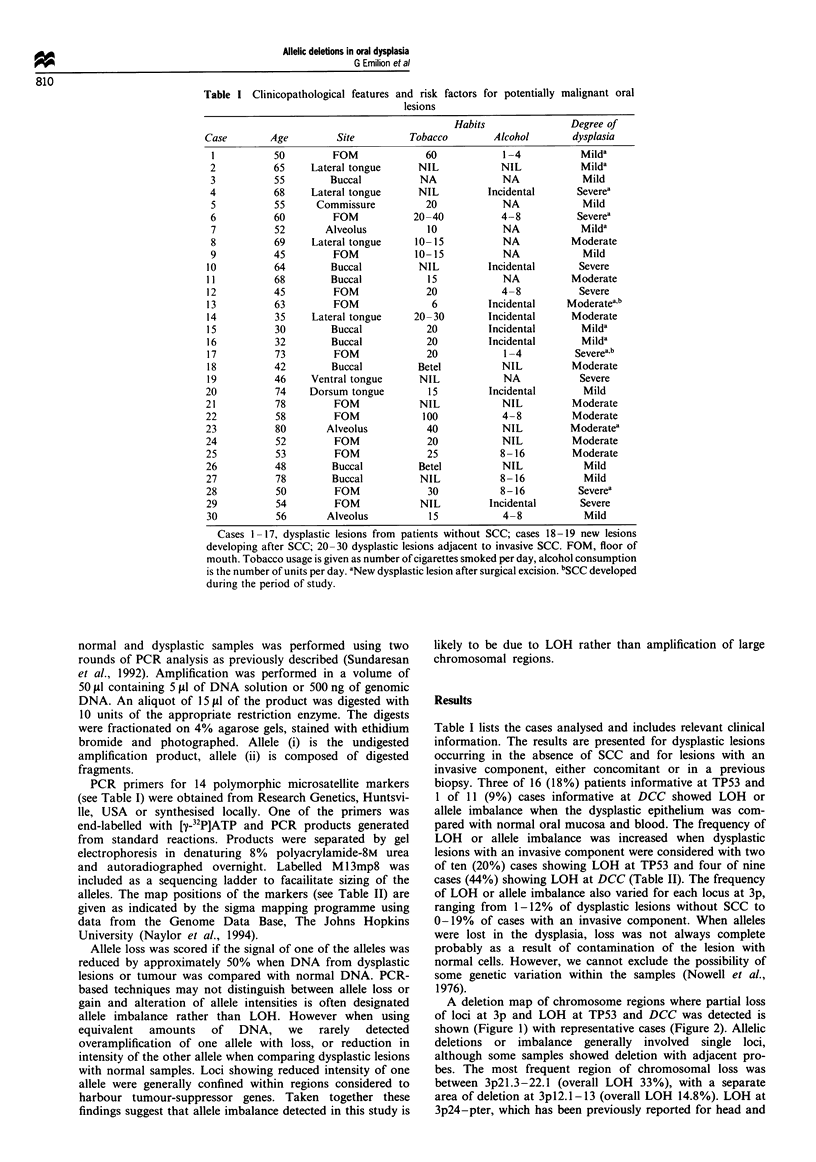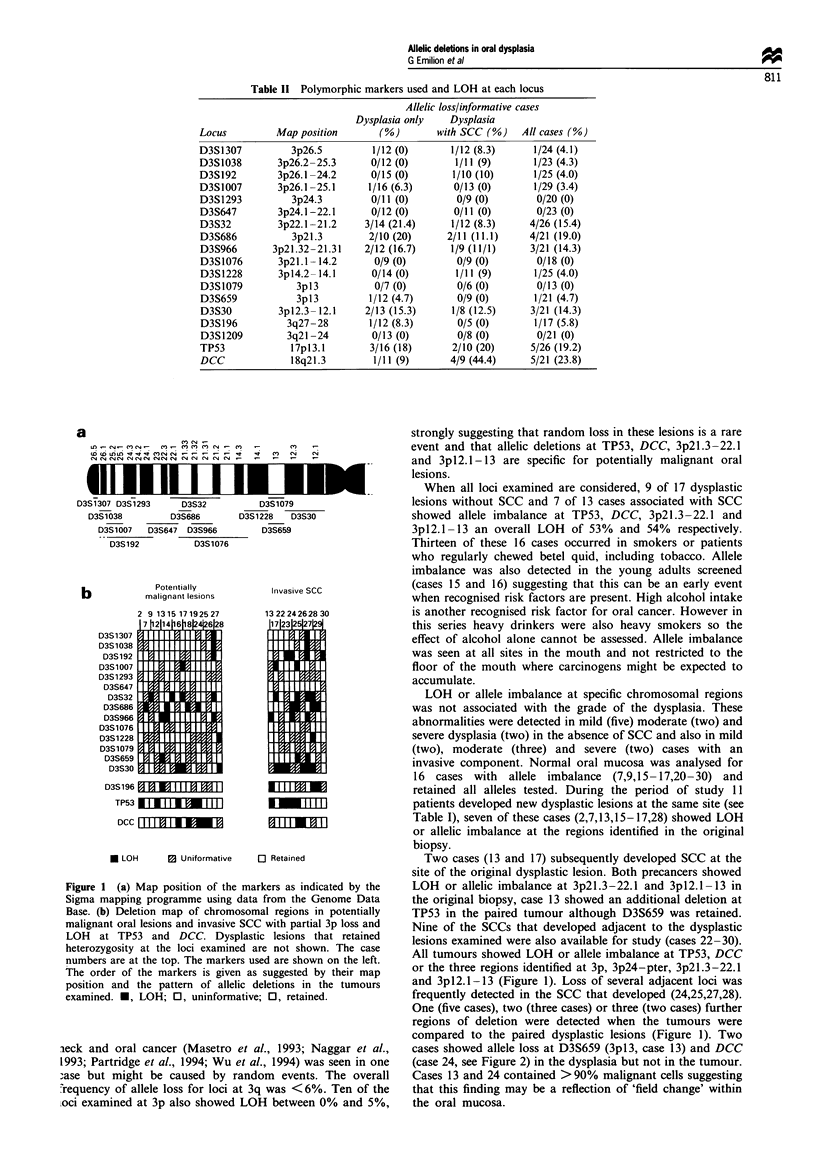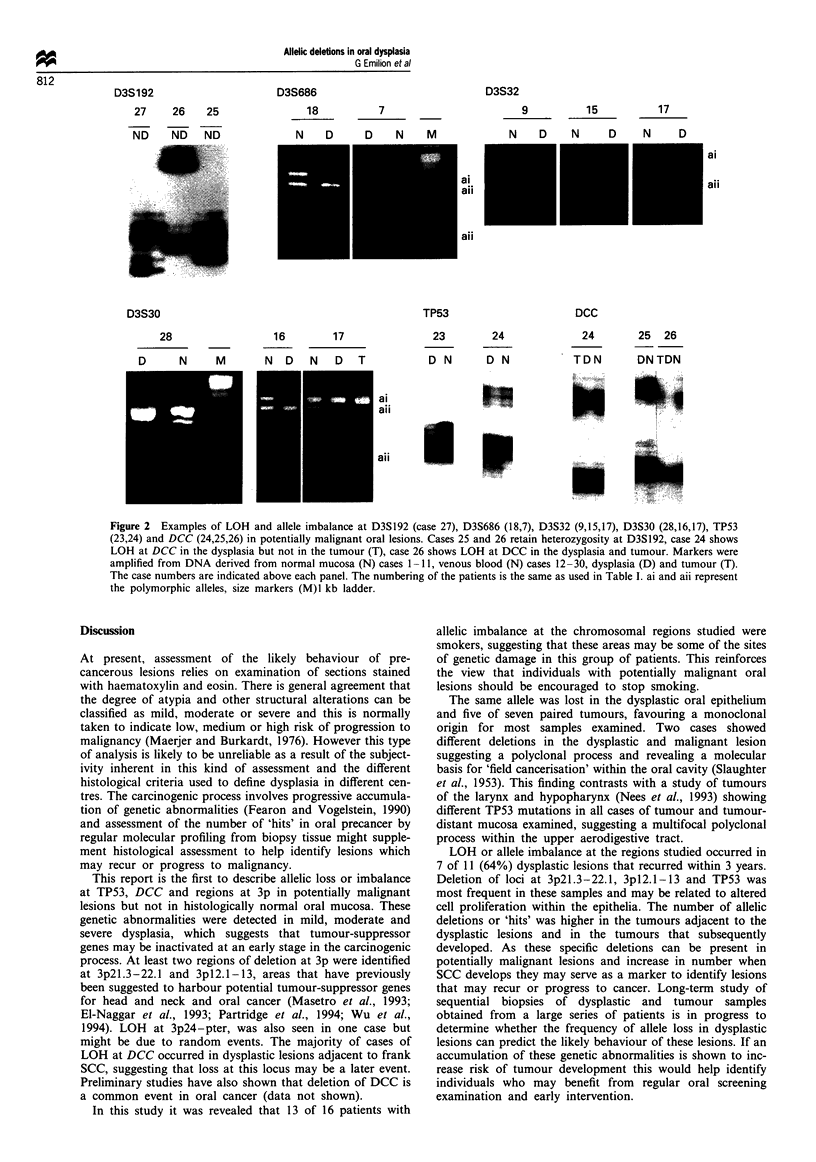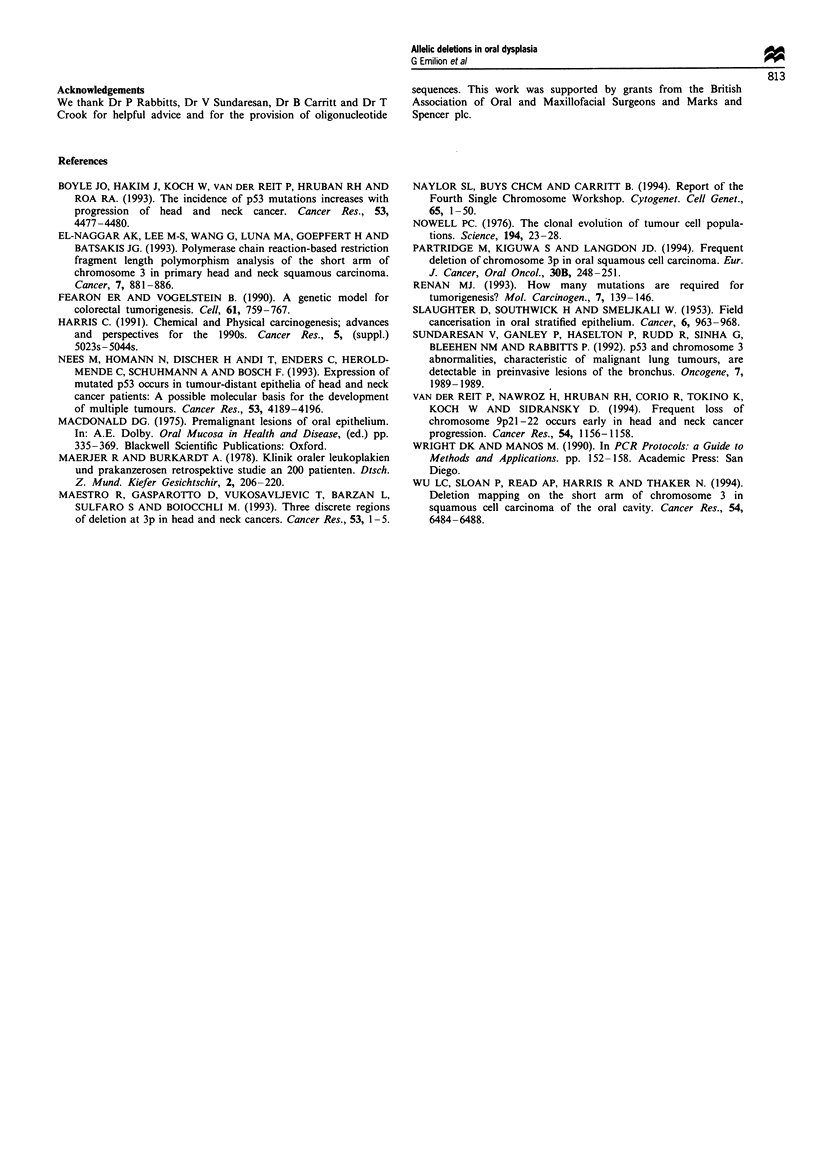# Frequent gene deletions in potentially malignant oral lesions.

**DOI:** 10.1038/bjc.1996.142

**Published:** 1996-03

**Authors:** G. Emilion, J. D. Langdon, P. Speight, M. Partridge

**Affiliations:** Department of Oral and Maxillofacial Surgery, King's College School of Medicine and Dentistry, London.

## Abstract

**Images:**


British Journal of Cancer (1996) 73, 809–813
© 1996 Stockton Press All rights reserved 0007–0920/96 $12.00

### Frequent gene deletions in potentially malignant oral lesions

G Emilion[1], JD Langdon[1], P Speight[2] and M Partridge[1]

[1]*Epithelial Cell Biology Unit, Department of Oral and Maxillofacial Surgery, King's College School of Medicine and Dentistry, Denmark Hill, London SE5 8RX;* [2]*Department of Oral Pathology, The Eastman Dental Institute, Gray's Inn Road, London WC1X 8LD.*

**Summary** Some oral cancers are preceded by premalignant lesions which include leucoplakia and erythroplakia. At present there are no reliable markers to identify lesions that may progress to malignancy. We have analysed 30 potentially malignant oral lesions for deletions at chromosomal regions that harbour tumour-suppressor genes for oral cancer. A total of 16 of 30 cases (53%) showed loss of heterozygosity (LOH) or allele imbalance at TP53, *DCC*, 3p21.3–22.1 or 3p12.1–13. These genetic alterations were detected in dysplastic lesions but not in histologically normal mucosa and may be early events in the carcinogenic process. A total of 64% of dysplastic lesions that recurred during the study showed LOH or allele imbalance in the initial biopsy and the number of genetic abnormalities increased in the tumours that developed. This type of molecular profiling may help to identify patients with lesions that may recur or acquire additional genetic events and progress to malignancy.

**Keywords:** chromosome deletion; gene; suppressor; tumour; mouth neoplasm

Early treatment of oral cancer offers the best chance of cure. However, patient awareness of this disease is low and as most cases present late treatment is associated with significant physical and psychological morbidity and reduced survival. There is increasing awareness of the potential value of screening for oral cancer and precancer to reduce morbidity and mortality. The most significant oral precancers are leucoplakia (a white patch) and erythroplakia (a red patch) which may show varying degrees of dysplasia. Longitudinal studies have shown that some of these lesions develop into invasive squamous cell carcinoma (SCC), although others regress. Depending on the population studied pre-existing leucoplakia, the most common oral premalignant condition, is associated with 16–32% of oral cancers (MacDonald, 1975). Erythroplakia is much rarer than leucoplakia but shows a much higher risk of malignant transformation. The clinical appearance of these lesions and recognised risk factors for oral cancer, which include heavy smoking and high alcohol consumption, are poorly predictive for risk of tumour development and therefore the lesions that will ultimately become neoplastic cannot readily be identified.

At present most clinicians biopsy all suspicious red and white patches treating only those showing severe dysplasia. Management of lesions showing mild or moderate dysplasia remains problematical as there are no markers that can predict risk of progression to malignancy. It is generally accepted that carcinogenesis involves the progressive accumulation of genetic abnormalities (Fearon and Vogelstein, 1990). Epidemiological, molecular and statistical studies have suggested that between six and ten genetic events are required for head and neck cancers (Harris, 1991; Renan 1993). Genetic abnormalities affecting tumour-suppressor genes including allele loss at 9p21–22 (van der Reit *et al.*, 1994) and TP53 mutations (Boyle *et al.*, 1993) have been detected in preinvasive head and neck squamous cell carcinoma (SCC) and provide insight into early critical events. Study of the number and nature of genetic abnormalities in potentially malignant lesions may supplement existing histological assessment and help predict the likely behaviour of these oral lesions and identify patients who may benefit from regular oral examination, preventative strategies and early treatment when necessary.

We have screened potentially malignant oral lesions, with evidence of dysplasia, for allele loss at TP53, *DCC* and for regions at 3p, which harbour suppressor genes for oral cancer. Normal mucosa was also examined to see whether allelic deletion can precede histological changes. We detected LOH or allele imbalance in the dysplastic lesions but not in histologically normal mucosa, suggesting that this can be an early event in the carcinogenic process. The frequency of these genetic abnormalities was higher in the tumours that developed. This suggests that specific deletions at TP53, *DCC* and 3p in potentially malignant lesions may identify lesions that acquire additional genetic alterations and progress to cancer.

Correspondence: M Partridge
Received 22 May 1995; revised 24 July 1995; accepted 8 August 1995

### Materials and methods

To obtain dysplastic tissue we biopsied areas of leucoplakia and erythroplakia that were surgically excised, occurring in the absence of frank carcinoma (17 cases). We also biopsied areas adjacent to frank SCC (11 cases). Two other samples were new dysplastic lesions developing following treatment of a primary SCC. All cases were followed for a minimum period of 36 months. Eleven patients developed a further suspicious lesion, with evidence of dysplasia at the same site or close to the original biopsy. Two patients subsequently developed SCC within 18 months (see Table I).

The majority of each sample was snap frozen in liquid nitrogen and stored at −70°C. A portion of each biopsy was fixed in formalin and processed for routine histopathological examination. The degree of dysplasia ranged from mild (12 cases), moderate (ten) to severe (eight). Venous blood was stored in sodium chloride EDTA tubes and kept at −20°C until required. In some cases histologically normal mucosa, from the margin of a biopsy (cases 1–11) or obtained from the opposite side of the mouth (cases 12–30), was available as a further control.

Frozen sections (10 μ) were mounted onto microscope slides with double-sided sticky tape and stained with toluidine blue. The normal epithelium, dysplastic epithelium and tumour were microdissected from the slide and digested in 50 μl of lysis buffer (50 mM TRIS pH 8.3, 1 mM EDTA, 0.5% Tween 20, 500 μg $ml^{-1}$ proteinase K (Wright and Manos, 1990). After incubation at 55°C overnight the protease was inactivated by 10 min at 95°C. Polymerase chain reaction (PCR) was performed directly on these aliquots. Genomic DNA was extracted from venous blood by lysis with Triton-X100.

To examine LOH at D3S686, D3S32 and D3S30 PCR-restriction fragment length polymorphism (RFLP) analysis of

**Table I** Clinicopathological features and risk factors for potentially malignant oral lesions

| Case | Age | Site | Habits: Tobacco | Habits: Alcohol | Degree of dysplasia |
|---|---|---|---|---|---|
| 1 | 50 | FOM | 60 | 1-4 | Mild[a] |
| 2 | 65 | Lateral tongue | NIL | NIL | Mild[a] |
| 3 | 55 | Buccal | NA | NA | Mild |
| 4 | 68 | Lateral tongue | NIL | Incidental | Severe[a] |
| 5 | 55 | Commissure | 20 | NA | Mild |
| 6 | 60 | FOM | 20-40 | 4-8 | Severe[a] |
| 7 | 52 | Alveolus | 10 | NA | Mild[a] |
| 8 | 69 | Lateral tongue | 10-15 | NA | Moderate |
| 9 | 45 | FOM | 10-15 | NA | Mild |
| 10 | 64 | Buccal | NIL | Incidental | Severe |
| 11 | 68 | Buccal | 15 | NA | Moderate |
| 12 | 45 | FOM | 20 | 4-8 | Severe |
| 13 | 63 | FOM | 6 | Incidental | Moderate[a,b] |
| 14 | 35 | Lateral tongue | 20-30 | Incidental | Moderate |
| 15 | 30 | Buccal | 20 | Incidental | Mild[a] |
| 16 | 32 | Buccal | 20 | Incidental | Mild[a] |
| 17 | 73 | FOM | 20 | 1-4 | Severe[a,b] |
| 18 | 42 | Buccal | Betel | NIL | Moderate |
| 19 | 46 | Ventral tongue | NIL | NA | Severe |
| 20 | 74 | Dorsum tongue | 15 | Incidental | Mild |
| 21 | 78 | FOM | NIL | NIL | Moderate |
| 22 | 58 | FOM | 100 | 4-8 | Moderate |
| 23 | 80 | Alveolus | 40 | NIL | Moderate[a] |
| 24 | 52 | FOM | 20 | NIL | Moderate |
| 25 | 53 | FOM | 25 | 8-16 | Moderate |
| 26 | 48 | Buccal | Betel | NIL | Mild |
| 27 | 78 | Buccal | NIL | 8-16 | Mild |
| 28 | 50 | FOM | 30 | 8-16 | Severe[a] |
| 29 | 54 | FOM | NIL | Incidental | Severe |
| 30 | 56 | Alveolus | 15 | 4-8 | Mild |

Cases 1-17, dysplastic lesions from patients without SCC; cases 18-19 new lesions developing after SCC; 20-30 dysplastic lesions adjacent to invasive SCC. FOM, floor of mouth. Tobacco usage is given as number of cigarettes smoked per day, alcohol consumption is the number of units per day. [a]New dysplastic lesion after surgical excision. [b]SCC developed during the period of study.

normal and dysplastic samples was performed using two rounds of PCR analysis as previously described (Sundaresan *et al.*, 1992). Amplification was performed in a volume of 50 μl containing 5 μl of DNA solution or 500 ng of genomic DNA. An aliquot of 15 μl of the product was digested with 10 units of the appropriate restriction enzyme. The digests were fractionated on 4% agarose gels, stained with ethidium bromide and photographed. Allele (i) is the undigested amplification product, allele (ii) is composed of digested fragments.

PCR primers for 14 polymorphic microsatellite markers (see Table I) were obtained from Research Genetics, Huntsville, USA or synthesised locally. One of the primers was end-labelled with [γ-$^{32}$P]ATP and PCR products generated from standard reactions. Products were separated by gel electrophoresis in denaturing 8% polyacrylamide-8M urea and autoradiographed overnight. Labelled M13mp8 was included as a sequencing ladder to facailitate sizing of the alleles. The map positions of the markers (see Table II) are given as indicated by the sigma mapping programme using data from the Genome Data Base, The Johns Hopkins University (Naylor *et al.*, 1994).

Allele loss was scored if the signal of one of the alleles was reduced by approximately 50% when DNA from dysplastic lesions or tumour was compared with normal DNA. PCR-based techniques may not distinguish between allele loss or gain and alteration of allele intensities is often designated allele imbalance rather than LOH. However when using equivalent amounts of DNA, we rarely detected overamplification of one allele with loss, or reduction in intensity of the other allele when comparing dysplastic lesions with normal samples. Loci showing reduced intensity of one allele were generally confined within regions considered to harbour tumour-suppressor genes. Taken together these findings suggest that allele imbalance detected in this study is likely to be due to LOH rather than amplification of large chromosomal regions.

### Results

Table I lists the cases analysed and includes relevant clinical information. The results are presented for dysplastic lesions occurring in the absence of SCC and for lesions with an invasive component, either concomitant or in a previous biopsy. Three of 16 (18%) patients informative at TP53 and 1 of 11 (9%) cases informative at *DCC* showed LOH or allele imbalance when the dysplastic epithelium was compared with normal oral mucosa and blood. The frequency of LOH or allele imbalance was increased when dysplastic lesions with an invasive component were considered with two of ten (20%) cases showing LOH at TP53 and four of nine cases (44%) showing LOH at *DCC* (Table II). The frequency of LOH or allele imbalance also varied for each locus at 3p, ranging from 1-12% of dysplastic lesions without SCC to 0-19% of cases with an invasive component. When alleles were lost in the dysplasia, loss was not always complete probably as a result of contamination of the lesion with normal cells. However, we cannot exclude the possibility of some genetic variation within the samples (Nowell *et al.*, 1976).

A deletion map of chromosome regions where partial loss of loci at 3p and LOH at TP53 and *DCC* was detected is shown (Figure 1) with representative cases (Figure 2). Allelic deletions or imbalance generally involved single loci, although some samples showed deletion with adjacent probes. The most frequent region of chromosomal loss was between 3p21.3-22.1 (overall LOH 33%), with a separate area of deletion at 3p12.1-13 (overall LOH 14.8%). LOH at 3p24-pter, which has been previously reported for head and

**Table II** Polymorphic markers used and LOH at each locus

| *Locus* | *Map position* | *Allelic loss/informative cases* Dysplasia only (%) | Dysplasia with SCC (%) | All cases (%) |
|---|---|---|---|---|
| D3S1307 | 3p26.5 | 1/12 (0) | 1/12 (8.3) | 1/24 (4.1) |
| D3S1038 | 3p26.2–25.3 | 0/12 (0) | 1/11 (9) | 1/23 (4.3) |
| D3S192 | 3p26.1–24.2 | 0/15 (0) | 1/10 (10) | 1/25 (4.0) |
| D3S1007 | 3p26.1–25.1 | 1/16 (6.3) | 0/13 (0) | 1/29 (3.4) |
| D3S1293 | 3p24.3 | 0/11 (0) | 0/9 (0) | 0/20 (0) |
| D3S647 | 3p24.1–22.1 | 0/12 (0) | 0/11 (0) | 0/23 (0) |
| D3S32 | 3p22.1–21.2 | 3/14 (21.4) | 1/12 (8.3) | 4/26 (15.4) |
| D3S686 | 3p21.3 | 2/10 (20) | 2/11 (11.1) | 4/21 (19.0) |
| D3S966 | 3p21.32–21.31 | 2/12 (16.7) | 1/9 (11/1) | 3/21 (14.3) |
| D3S1076 | 3p21.1–14.2 | 0/9 (0) | 0/9 (0) | 0/18 (0) |
| D3S1228 | 3p14.2–14.1 | 0/14 (0) | 1/11 (9) | 1/25 (4.0) |
| D3S1079 | 3p13 | 0/7 (0) | 0/6 (0) | 0/13 (0) |
| D3S659 | 3p13 | 1/12 (4.7) | 0/9 (0) | 1/21 (4.7) |
| D3S30 | 3p12.3–12.1 | 2/13 (15.3) | 1/8 (12.5) | 3/21 (14.3) |
| D3S196 | 3q27–28 | 1/12 (8.3) | 0/5 (0) | 1/17 (5.8) |
| D3S1209 | 3q21–24 | 0/13 (0) | 0/8 (0) | 0/21 (0) |
| TP53 | 17p13.1 | 3/16 (18) | 2/10 (20) | 5/26 (19.2) |
| *DCC* | 18q21.3 | 1/11 (9) | 4/9 (44.4) | 5/21 (23.8) |

**Figure 1** (**a**) Map position of the markers as indicated by the Sigma mapping programme using data from the Genome Data Base. (**b**) Deletion map of chromosomal regions in potentially malignant oral lesions and invasive SCC with partial 3p loss and LOH at TP53 and *DCC*. Dysplastic lesions that retained heterozygosity at the loci examined are not shown. The case numbers are at the top. The markers used are shown on the left. The order of the markers is given as suggested by their map position and the pattern of allelic deletions in the tumours examined. ■, LOH; □, uninformative; □, retained.

neck and oral cancer (Masetro *et al*., 1993; Naggar *et al*., 1993; Partridge *et al*., 1994; Wu *et al*., 1994) was seen in one case but might be caused by random events. The overall frequency of allele loss for loci at 3q was <6%. Ten of the loci examined at 3p also showed LOH between 0% and 5%, strongly suggesting that random loss in these lesions is a rare event and that allelic deletions at TP53, *DCC*, 3p21.3–22.1 and 3p12.1–13 are specific for potentially malignant oral lesions.

When all loci examined are considered, 9 of 17 dysplastic lesions without SCC and 7 of 13 cases associated with SCC showed allele imbalance at TP53, *DCC*, 3p21.3–22.1 and 3p12.1–13 an overall LOH of 53% and 54% respectively. Thirteen of these 16 cases occurred in smokers or patients who regularly chewed betel quid, including tobacco. Allele imbalance was also detected in the young adults screened (cases 15 and 16) suggesting that this can be an early event when recognised risk factors are present. High alcohol intake is another recognised risk factor for oral cancer. However in this series heavy drinkers were also heavy smokers so the effect of alcohol alone cannot be assessed. Allele imbalance was seen at all sites in the mouth and not restricted to the floor of the mouth where carcinogens might be expected to accumulate.

LOH or allele imbalance at specific chromosomal regions was not associated with the grade of the dysplasia. These abnormalities were detected in mild (five) moderate (two) and severe dysplasia (two) in the absence of SCC and also in mild (two), moderate (three) and severe (two) cases with an invasive component. Normal oral mucosa was analysed for 16 cases with allele imbalance (7,9,15–17,20–30) and retained all alleles tested. During the period of study 11 patients developed new dysplastic lesions at the same site (see Table I), seven of these cases (2,7,13,15–17,28) showed LOH or allelic imbalance at the regions identified in the original biopsy.

Two cases (13 and 17) subsequently developed SCC at the site of the original dysplastic lesion. Both precancers showed LOH or allelic imbalance at 3p21.3–22.1 and 3p12.1–13 in the original biopsy, case 13 showed an additional deletion at TP53 in the paired tumour although D3S659 was retained. Nine of the SCCs that developed adjacent to the dysplastic lesions examined were also available for study (cases 22–30). All tumours showed LOH or allele imbalance at TP53, *DCC* or the three regions identified at 3p, 3p24–pter, 3p21.3–22.1 and 3p12.1–13 (Figure 1). Loss of several adjacent loci was frequently detected in the SCC that developed (24,25,27,28). One (five cases), two (three cases) or three (two cases) further regions of deletion were detected when the tumours were compared to the paired dysplastic lesions (Figure 1). Two cases showed allele loss at D3S659 (3p13, case 13) and *DCC* (case 24, see Figure 2) in the dysplasia but not in the tumour. Cases 13 and 24 contained >90% malignant cells suggesting that this finding may be a reflection of 'field change' within the oral mucosa.

**Figure 2** Examples of LOH and allele imbalance at D3S192 (case 27), D3S686 (18,7), D3S32 (9,15,17), D3S30 (28,16,17), TP53 (23,24) and *DCC* (24,25,26) in potentially malignant oral lesions. Cases 25 and 26 retain heterozygosity at D3S192, case 24 shows LOH at *DCC* in the dysplasia but not in the tumour (T), case 26 shows LOH at DCC in the dysplasia and tumour. Markers were amplified from DNA derived from normal mucosa (N) cases 1–11, venous blood (N) cases 12–30, dysplasia (D) and tumour (T). The case numbers are indicated above each panel. The numbering of the patients is the same as used in Table I. ai and aii represent the polymorphic alleles, size markers (M)1 kb ladder.

### Discussion

At present, assessment of the likely behaviour of precancerous lesions relies on examination of sections stained with haematoxylin and eosin. There is general agreement that the degree of atypia and other structural alterations can be classified as mild, moderate or severe and this is normally taken to indicate low, medium or high risk of progression to malignancy (Maerjer and Burkardt, 1976). However this type of analysis is likely to be unreliable as a result of the subjectivity inherent in this kind of assessment and the different histological criteria used to define dysplasia in different centres. The carcinogenic process involves progressive accumulation of genetic abnormalities (Fearon and Vogelstein, 1990) and assessment of the number of 'hits' in oral precancer by regular molecular profiling from biopsy tissue might supplement histological assessment to help identify lesions which may recur or progress to malignancy.

This report is the first to describe allelic loss or imbalance at TP53, *DCC* and regions at 3p in potentially malignant lesions but not in histologically normal oral mucosa. These genetic abnormalities were detected in mild, moderate and severe dysplasia, which suggests that tumour-suppressor genes may be inactivated at an early stage in the carcinogenic process. At least two regions of deletion at 3p were identified at 3p21.3–22.1 and 3p12.1–13, areas that have previously been suggested to harbour potential tumour-suppressor genes for head and neck and oral cancer (Masetro *et al.*, 1993; El-Naggar *et al.*, 1993; Partridge *et al.*, 1994; Wu *et al.*, 1994). LOH at 3p24–pter, was also seen in one case but might be due to random events. The majority of cases of LOH at *DCC* occurred in dysplastic lesions adjacent to frank SCC, suggesting that loss at this locus may be a later event. Preliminary studies have also shown that deletion of DCC is a common event in oral cancer (data not shown).

In this study it was revealed that 13 of 16 patients with allelic imbalance at the chromosomal regions studied were smokers, suggesting that these areas may be some of the sites of genetic damage in this group of patients. This reinforces the view that individuals with potentially malignant oral lesions should be encouraged to stop smoking.

The same allele was lost in the dysplastic oral epithelium and five of seven paired tumours, favouring a monoclonal origin for most samples examined. Two cases showed different deletions in the dysplastic and malignant lesion suggesting a polyclonal process and revealing a molecular basis for 'field cancerisation' within the oral cavity (Slaughter *et al.*, 1953). This finding contrasts with a study of tumours of the larynx and hypopharynx (Nees *et al.*, 1993) showing different TP53 mutations in all cases of tumour and tumour-distant mucosa examined, suggesting a multifocal polyclonal process within the upper aerodigestive tract.

LOH or allele imbalance at the regions studied occurred in 7 of 11 (64%) dysplastic lesions that recurred within 3 years. Deletion of loci at 3p21.3–22.1, 3p12.1–13 and TP53 was most frequent in these samples and may be related to altered cell proliferation within the epithelia. The number of allelic deletions or 'hits' was higher in the tumours adjacent to the dysplastic lesions and in the tumours that subsequently developed. As these specific deletions can be present in potentially malignant lesions and increase in number when SCC develops they may serve as a marker to identify lesions that may recur or progress to cancer. Long-term study of sequential biopsies of dysplastic and tumour samples obtained from a large series of patients is in progress to determine whether the frequency of allele loss in dysplastic lesions can predict the likely behaviour of these lesions. If an accumulation of these genetic abnormalities is shown to increase risk of tumour development this would help identify individuals who may benefit from regular oral screening examination and early intervention.

**Acknowledgements**
We thank Dr P Rabbitts, Dr V Sundaresan, Dr B Carritt and Dr T Crook for helpful advice and for the provision of oligonucleotide sequences. This work was supported by grants from the British Association of Oral and Maxillofacial Surgeons and Marks and Spencer plc.

### References

BOYLE JO, HAKIM J, KOCH W, VAN DER REIT P, HRUBAN RH AND ROA RA. (1993). The incidence of p53 mutations increases with progression of head and neck cancer. *Cancer Res.*, **53**, 4477–4480.

EL-NAGGAR AK, LEE M-S, WANG G, LUNA MA, GOEPFERT H AND BATSAKIS JG. (1993). Polymerase chain reaction-based restriction fragment length polymorphism analysis of the short arm of chromosome 3 in primary head and neck squamous carcinoma. *Cancer*, **7**, 881–886.

FEARON ER AND VOGELSTEIN B. (1990). A genetic model for colorectal tumorigenesis. *Cell*, **61**, 759–767.

HARRIS C. (1991). Chemical and Physical carcinogenesis; advances and perspectives for the 1990s. *Cancer Res.*, **5**, (suppl.) 5023s–5044s.

NEES M, HOMANN N, DISCHER H ANDI T, ENDERS C, HEROLD-MENDE C, SCHUHMANN A AND BOSCH F. (1993). Expression of mutated p53 occurs in tumour-distant epithelia of head and neck cancer patients: A possible molecular basis for the development of multiple tumours. *Cancer Res.*, **53**, 4189–4196.

MACDONALD DG. (1975). Premalignant lesions of oral epithelium. In: A.E. Dolby. *Oral Mucosa in Health and Disease*, (ed.) pp. 335–369. Blackwell Scientific Publications: Oxford.

MAERJER R AND BURKARDT A. (1978). Klinik oraler leukoplakien und prakanzerosen retrospektive studie an 200 patienten. *Dtsch. Z. Mund. Kiefer Gesichtschir*, **2**, 206–220.

MAESTRO R, GASPAROTTO D, VUKOSAVLJEVIC T, BARZAN L, SULFARO S AND BOIOCCHLI M. (1993). Three discrete regions of deletion at 3p in head and neck cancers. *Cancer Res.*, **53**, 1–5.

NAYLOR SL, BUYS CHCM AND CARRITT B. (1994). Report of the Fourth Single Chromosome Workshop. *Cytogenet. Cell Genet.*, **65**, 1–50.

NOWELL PC. (1976). The clonal evolution of tumour cell populations. *Science*, **194**, 23–28.

PARTRIDGE M, KIGUWA S AND LANGDON JD. (1994). Frequent deletion of chromosome 3p in oral squamous cell carcinoma. *Eur. J. Cancer, Oral Oncol.*, **30B**, 248–251.

RENAN MJ. (1993). How many mutations are required for tumorigenesis? *Mol. Carcinogen.*, **7**, 139–146.

SLAUGHTER D, SOUTHWICK H AND SMELJKALI W. (1953). Field cancerisation in oral stratified epithelium. *Cancer*, **6**, 963–968.

SUNDARESAN V, GANLEY P, HASELTON P, RUDD R, SINHA G, BLEEHEN NM AND RABBITTS P. (1992). p53 and chromosome 3 abnormalities, characteristic of malignant lung tumours, are detectable in preinvasive lesions of the bronchus. *Oncogene*, **7**, 1989–1989.

VAN DER REIT P, NAWROZ H, HRUBAN RH, CORIO R, TOKINO K, KOCH W AND SIDRANSKY D. (1994). Frequent loss of chromosome 9p21–22 occurs early in head and neck cancer progression. *Cancer Res.*, **54**, 1156–1158.

WRIGHT DK AND MANOS M. (1990). In *PCR Protocols: a Guide to Methods and Applications*. pp. 152–158. Academic Press: San Diego.

WU LC, SLOAN P, READ AP, HARRIS R AND THAKER N. (1994). Deletion mapping on the short arm of chromosome 3 in squamous cell carcinoma of the oral cavity. *Cancer Res.*, **54**, 6484–6488.